# *Ex vivo* evaluation of the Ozaki procedure in comparison with the native aortic valve and prosthetic valves

**DOI:** 10.1093/icvts/ivac199

**Published:** 2022-07-27

**Authors:** Hiroyuki Saisho, Michael Scharfschwerdt, Tim Schaller, Najla Sadat, Anas Aboud, Stephan Ensminger, Buntaro Fujita

**Affiliations:** Department of Cardiac and Thoracic Vascular Surgery, University Hospital of Schleswig Holstein, Lübeck, Germany; University of Lübeck, Lübeck, Germany; Department of Cardiac and Thoracic Vascular Surgery, University Hospital of Schleswig Holstein, Lübeck, Germany; University of Lübeck, Lübeck, Germany; Department of Cardiac and Thoracic Vascular Surgery, University Hospital of Schleswig Holstein, Lübeck, Germany; University of Lübeck, Lübeck, Germany; Department of Cardiac and Thoracic Vascular Surgery, University Hospital of Schleswig Holstein, Lübeck, Germany; University of Lübeck, Lübeck, Germany; DZHK (German Centre for Cardiovascular Research—Partner Site Hamburg/Kiel/Lübeck), Germany; Department of Cardiac and Thoracic Vascular Surgery, University Hospital of Schleswig Holstein, Lübeck, Germany; University of Lübeck, Lübeck, Germany; Department of Cardiac and Thoracic Vascular Surgery, University Hospital of Schleswig Holstein, Lübeck, Germany; University of Lübeck, Lübeck, Germany; DZHK (German Centre for Cardiovascular Research—Partner Site Hamburg/Kiel/Lübeck), Germany; Department of Cardiac and Thoracic Vascular Surgery, University Hospital of Schleswig Holstein, Lübeck, Germany; University of Lübeck, Lübeck, Germany; DZHK (German Centre for Cardiovascular Research—Partner Site Hamburg/Kiel/Lübeck), Germany

**Keywords:** Ozaki procedure, Aortic valve replacement, Ex vivo, Valve motion, Hydrodynamic performance

## Abstract

**OBJECTIVES:**

We investigated the hydrodynamic performance and cusp kinematics of the Ozaki neocuspidized aortic valve in comparison with the native aortic and prosthetic valves in an *ex vivo* study.

**METHODS:**

Native aortic valves of swine hearts were replaced by aortic valve substitutes, and their hydrodynamic performance (effective orifice area and mean pressure gradient) was evaluated in a mock circulation under defined conditions. The following aortic valve substitutes were investigated: native aortic valve, Ozaki valve, Perimount Magna Ease, Trifecta and St. Jude Medical Masters. All prosthetic valves had a labelled size of 21 mm.

**RESULTS:**

The Ozaki valve and native aortic valve showed a similar and significantly larger orifice area than all investigated prosthetic valves particularly at high flow rates. There was no significant difference between the Ozaki valve and the native aortic valve. The native aortic valve and Ozaki valve showed a similar increase in orifice area with increasing flow through the valve while prosthetic valves showed a markedly weaker increase. Similarly, the native and Ozaki valve showed a similar increase in mPG with forward flow which was weaker than prosthetic valves. Cusp kinematics were similar between the native and Ozaki valve, whilst prosthetic valves were clearly distinguishable from them.

**CONCLUSIONS:**

The Ozaki procedure showed excellent hydrodynamic performance compared to prosthetic valves and showed similar cusp motion characteristics to the native aortic valve. Our results suggest that the Ozaki neocuspidized valve behaves physiologically in many aspects, which may contribute to beneficial clinical outcomes.

**Figure:**
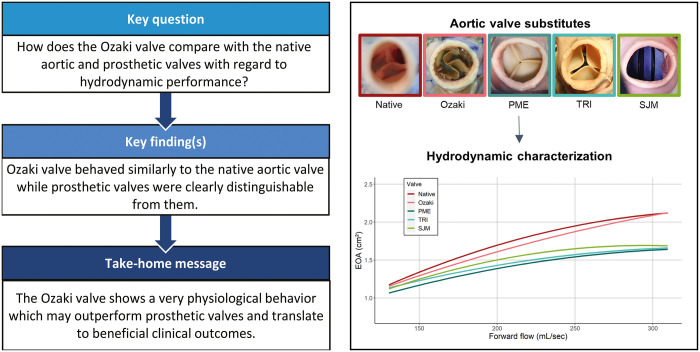

## INTRODUCTION

Treatment of aortic stenosis (AS) in non-elderly patients poses a tremendous clinical challenge. While surgical aortic valve replacement is the treatment modality of choice, there is an ongoing debate on the preferred valve substitute in these patients [1–4]. Even though multiple reports suggest that the Ross procedure achieves the best long-term results, this procedure lacks general acceptance by the medical community, which is in part related to the complexity of the procedure [5]. Therefore, mechanical valves have been the prosthesis of choice to avoid redo surgery, which is very likely for young patients who receive a bioprosthesis. However, this occurs at the cost of lifelong anticoagulation and its associated risks. Importantly, neither mechanical nor bioprosthetic valves are able to restore life expectancy of the general population [6, 7]. Furthermore, it is generally accepted that neither mechanical nor bioprosthetic valves can restore physiological flow conditions over the aortic valve. To overcome these limitations, an aortic valve neocuspidization procedure using autologous pericardium (the Ozaki procedure) has been introduced [8]. Ozaki *et al.* [9] reported good clinical results with excellent early- and mid-term mortality, low rates of redo surgeries and excellent haemodynamic performance. The Ozaki procedure offers some theoretical advantages that may contribute to long-term durability such as the geometrical shape of the neocusps, the implantation technique, a large effective orifice area (EOA) and preservation of aortic annular elasticity. These features distinguish the Ozaki procedure from conventional prosthetic valves. However, the Ozaki procedure has not been thoroughly investigated regarding these issues and has also not been directly compared to conventional prosthetic valves. Accordingly, the aim of the study was to investigate the hydrodynamic performance and cusp motion characteristics of a neocuspidized valve in comparison with the native aortic valve and established prosthetic valves in an *ex vivo* setting using a custom-made mock circulation loop.

## MATERIALS AND METHODS

### Ethics statement

The study was conducted in accordance with the German Animal Welfare Act and did not require approval by a local review board (TierSchG).

### Aortic root models

**Figure 1:**
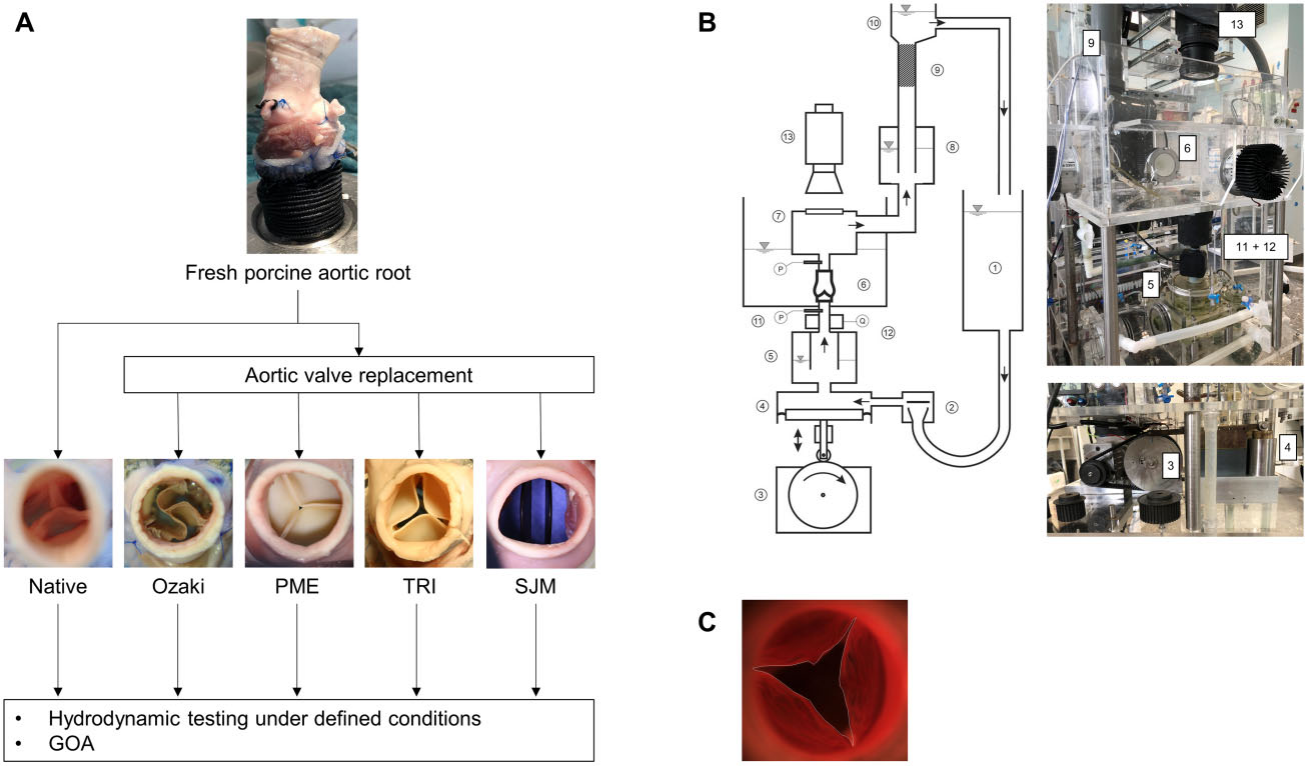
Study design and methods. (**A**) Flow chart of the investigated experimental groups. A fresh native porcine aortic root was dissected and connected to a Dacron prosthesis at the left ventricular outflow tract and the coronary arteries were ligated. Surgical aortic valve replacement was performed using 4 substitutes: the Ozaki valve, Perimount Magna Ease (PME) 21 mm, Trifecta (TRI) 21 mm and Masters mechanical heart valve (SJM) 21 mm. (**B**) Schematic depiction (left) and photographs (right) of the custom built mock circulation loop including the pulse duplicator (modified with permission from Scharfschwerdt *et al.* [10]). (1) Atrial reservoir, (2) disc valve, (3) cam plate, (4) piston pump, (5) adjustable input compliance, (6) fluid reservoir with an interchangeable aortic valve compartment, (7) visualization chamber, (8) adjustable aortic compliance, (9) non-linear resistance element, (10) height variable fluid column, (11) pressure sensor, (12) ultrasonic flow probe and (13) high-speed camera. (**C**) Exemplary illustration of GOA tracing.

Because of their similar anatomy compared to human aortic roots, porcine aortic roots were chosen for this *ex vivo* model. The ascending aorta and the left ventricular outflow tract were dissected, and the coronary arteries were ligated shortly after their origin from the aortic root. Short pieces of Dacron prostheses were sewn on the left ventricular outflow tract to place the aortic root models into a custom-made mock circulation loop. The native aortic valve was surgically replaced by different aortic valve substitutes (Fig. 1A). The Ozaki procedure was performed according to the protocol by Ozaki *et al.* [8] only with the difference that porcine instead of human pericardium was used. Briefly, the pericardium was treated with 0.625% glutaraldehyde and 3 neocusps were cut out using the original template after individual sizing of the 3 cusps. The cusps were sewn into the annulus as recommended by the protocol. In 7 cases, 3 mm × 23 mm cusps were used, while in the remaining 3 valves, 2 mm × 23 mm and 1 mm × 21 mm cusps were used. In addition, 3 widely used prosthetic valves were investigated: the Perimount Magna Ease (PME; Edwards Lifesciences LLC, Irvine, CA, USA), Trifecta (TRI) GT (Abbott, Chicago, IL, USA) and St. Jude Medical Masters HP™ (SJM) mechanical heart valve (St. Jude Medical Inc., St. Paul, MN, USA) all of which with a labelled size of 21 mm. The porcine hearts were deemed suitable for use in the study only once the aortic ring was measured with the corresponding sizer of each biological prosthesis and the result was 21 mm. Implantation of the prosthetic valves was performed in the usual manner using pledgeted 2/0 sutures and a non-everting suture technique. Therefore, a total of 5 groups were investigated: native aortic valve, Ozaki neocuspidized valve, PME, TRI and SJM.

### Physiological mock circulation

Physiological circulatory conditions were imitated by a pulse duplicator that can be adjusted to produce defined stroke volumes and heart rates under adjustable preloads and afterloads allowing for the evaluation of hydrodynamic valve function [10]. Stroke volume was generated by a piston pump which was displaced using a turning cam plate. The stroke volume can be modified while the duration of systole and the ratio systole:diastole (35%:65%) stay constant. Therefore, during higher stroke volumes, more volume per time unit passes through the aortic valve during systole. This volume per time was defined as ‘forward flow’ and given as ml/s for the present study.

For the present analysis, testing was performed under 4 hydrodynamic settings to simulate different cardiac outputs (i.e. ‘exercise’ levels). All conditions were run at a heart rate of 64/min and systemic pressure of 120/80 mmHg using physiological saline at 37°C:

Condition 1: stroke volume = 55.9±2.3 ml;
Condition 2: stroke volume = 73.3±2.9 ml;
Condition 3: stroke volume = 89.7±3.3 ml; and
Condition 4: stroke volume = 104.1±4.0 ml.

For all investigated aortic roots and all conditions, the EOA and mean pressure gradient (mPG) were determined.

The measurement of EOA and mPG was performed in accordance with the international norm for testing surgical prosthetic heart valves (ISO 5840: cardiovascular implants—cardiac valve prostheses) [11]. Briefly, the left ventricular pressure (4 cm below the aortic valve) and aortic pressure (6 cm above the aortic valve) were measured with 2 capacitive pressure transducers (Envec Ceracore M, Endress + Hauser, Maulburg, Germany), calibrated to a measuring range of −20 to +160 mmHg and a resolution of 0.02 mmHg. The sensor of an ultrasonic flowmeter (TS-410; Transonic System Inc., Ithaca, NY, USA) was mounted directly below the aortic valve to record the volume flow through the valve. The sensor works bidirectionally with a resolution of 2 ml/min and records flow rates up to 20 l/min.

### Assessment of the geometrical orifice area and its relation to the cardiac cycle

A high-speed motion camera (MotionPro Y3; Imaging Solutions GmbH, Eningen, Germany) mounted on top of the aortic root models enabled the capture of moving and still images of the aortic valve during opening and closing. Images were acquired at 500 frames/second. The geometric orifice area (GOA) was determined by tracing the orifice area at each frame throughout the entire cardiac cycle (Video 1). The GOA was depicted as a function of time within the cardiac cycle. In addition, flow through the valve was also depicted as a function of time. These 2 plots were overlaid to generate ‘GOA-flow-time plots’. This allowed us to investigate cusp motion characteristics with respect to the time of the cardiac cycle and flow through the valve.

### Statistical analysis

EOA and mPG were not normally distributed within most groups as assessed using Kolmogorov–Smirnoff test and visual inspection of histograms. All quantitative data are presented as median (25th–75th percentile). The Kruskal–Wallis test was used to test independent groups for differences. Tukey’s adjustment was used to adjust for multiple testing. A two-sided *P*-value of <0.05 was considered statistically significant. Polynomial regression was used to investigate the relationship between EOA, mPG and forward flow through the valve. Analysis of variance was applied to investigate whether the type of aortic valve had a significant effect. All statistical analyses were performed using SPSS version 26 (IBM, Armonk, NY, USA) and R version 4.0.2: A language and environment for statistical computing (R Foundation for Statistical Computing, Vienna, Austria).

## RESULTS

### Hydrodynamic performance

The native aortic valve and Ozaki valve showed the largest EOA and the lowest mPG under all conditions (Table 1). The native and Ozaki valve showed similar EOA as well as mPG compared with prosthetic valves with lower stroke volumes while EOA and mPG differed more often significantly with higher stroke volumes (Table 1 and Supplementary Material, Table 2).

**Table 1:** Hydrodynamic performance of investigated heart valves at various conditions

	Native	Ozaki	PME	TRI	SJM
	(*n* = 18)	(*n* = 10)	(*n* = 10)	(*n* = 10)	(*n* = 5)
EOA (cm^2^)					
Condition 1	1.33 (1.29–1.90)	1.41 (1.25–1.73)	1.28 (1.16–1.51)	1.19 (1.15–1.24)	1.27 (1.22–1.29)
Condition 2	1.58 (1.51–2.11)	1.60 (1.43–1.94)	1.42 (1.29–1.51)	1.37 (1.32–1.41)	1.44 (1.42–1.44)
Condition 3	1.70 (1.65–2.28)	1.70 (1.62–2.06)	1.49 (1.42–1.55)	1.49 (1.44–1.54)	1.59 (1.55–1.58)
Condition 4	1.88 (1.76–2.32)	1.86 (1.78–2.14)	1.54 (1.53–1.58)	1.62 (1.59–1.68)	1.64 (1.63–1.68)
mPG (mmHg)					
Condition 1	6.47 (4.12–7.48)	6.36 (5.45–7.41)	8.03 (7.00–8.79)	8.26 (7.17–8.85)	6.91 (6.72–7.78)
Condition 2	7.49 (4.85–8.20)	7.21 (5.90–8.16)	9.65 (8.29–10.40)	9.61 (8.58–10.30)	8.39 (7.53–8.91)
Condition 3	8.26 (5.40–9.27)	8.16 (6.75–9.33)	10.83 (10.17–12.36)	10.37 (9.92–12.13)	9.12 (8.71–9.59)
Condition 4	9.07 (7.05–10.55)	9.45 (7.57–10.36)	12.41 (11.36–13.88)	11.83 (11.45–13.28)	9.96 (9.24–10.26)

EOA: effective orifice area; mPG: mean pressure gradient; PME: Perimount Magna Ease; SJM: St. Jude Medical Masters HP; TRI: Trifecta.

**Figure 2:**
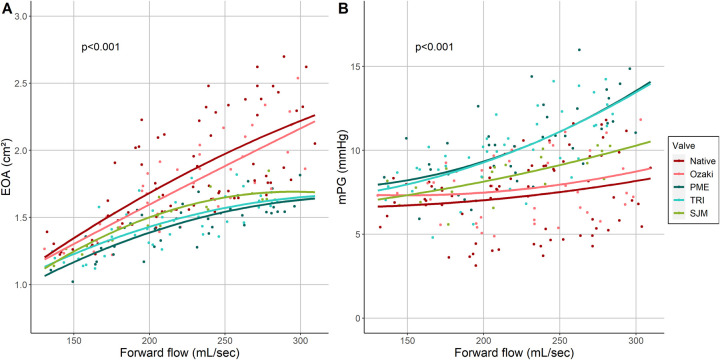
(**A**) Effective orifice area of the investigated aortic valves as a function of forward flow. Lines show quadratic regression lines. (**B**) Mean pressure gradient over the investigated aortic valves as a function of forward flow. Lines show quadratic regression lines.

All valves showed an increase in EOA with increasing forward flow through the valve but the valve had a significant effect (*P* < 0.001) (Fig. 2A). The native aortic valve showed the steepest increase of EOA with increasing forward flow, followed by the Ozaki valve. For these 2 valves, the rate of EOA increase was fairly constant over the investigated range of forward flow. On the contrary, all 3 prosthetic valves approached a plateau and started to divide from the native and Ozaki valve at approximately 200 ml/s.

Similarly, all valves showed an increase in mPG with increasing forward flow where the valve type had a significant effect (*P* < 0.001) (Fig. 2B). The native aortic valve and the Ozaki valve showed a similar course. SJM showed a stronger increase in mPG with forward flow while this response was strongest for the TRI and PME.

### Analysis of cusp motion—'GOA-flow-time plots’

**Figure 3:**
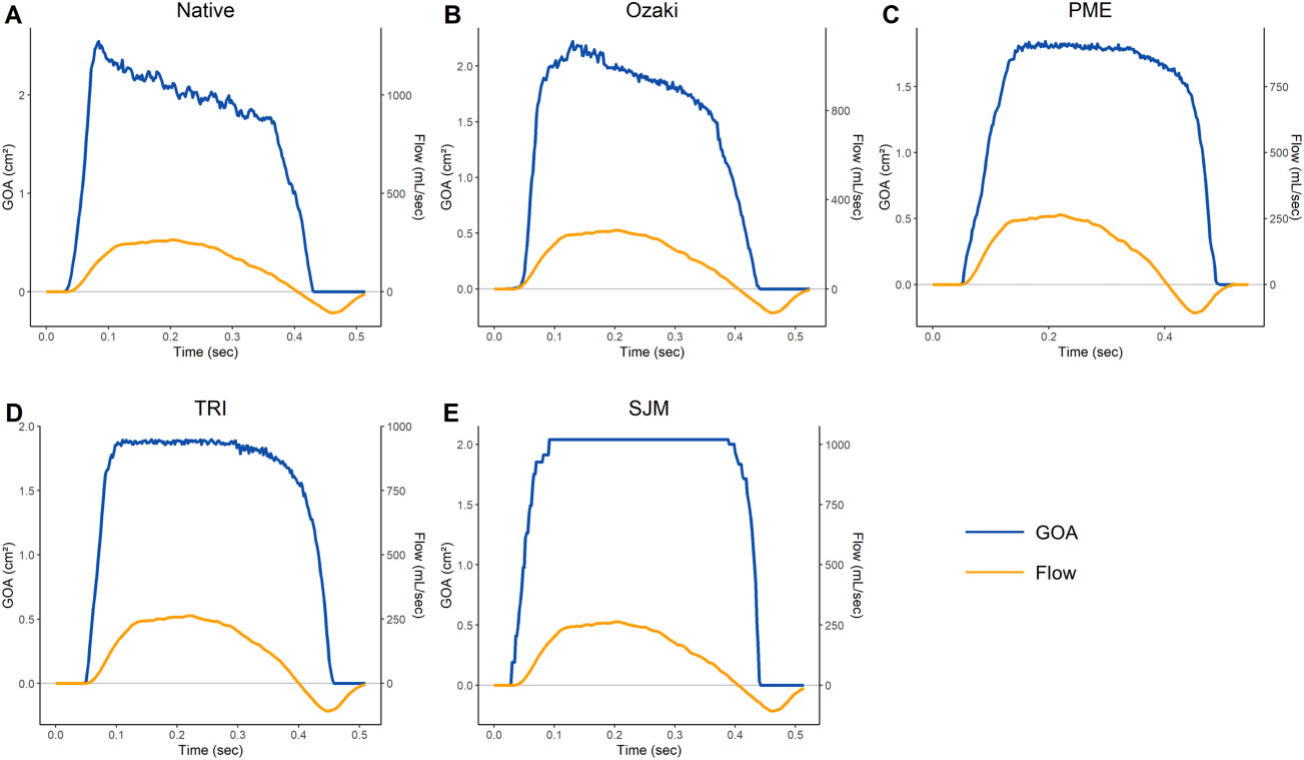
GOA-flow-time plots of (**A**) native aortic valve, (**B**) Ozaki valve, (**C**) Perimount Magna Ease, (**D**) Trifecta and (**E**) St. Jude Medical Masters HP.

The GOA and transvalvular flow as a function of time for all investigated valves are shown in Fig. 3. The native aortic valve showed a rapid initial opening phase until maximum opening followed by a gradual closure of the valve at a constant rate which started in early systole while forward flow through the valve was still increasing. This phase was followed by a rapid closure phase before full closure was achieved (Fig. 3A). The Ozaki valve showed a rapid initial opening phase, followed by a second and slower opening phase to reach maximum opening. The Ozaki valve then started a gradual and constant closure phase followed by rapid closure to achieve full closure (Fig. 3B). The PME showed an initial opening phase, which was not as steep as the native valve and the Ozaki valve to reach maximum opening followed by a constant GOA through most of the systolic phase. The valve then started to slowly close before entering a rapid closure phase (Fig. 3C). The TRI showed a rapid initial valve opening to maximum GOA which stayed constant throughout most of systole, before a slow closure phase followed by rapid closure of the valve (Fig. 3D). The SJM rapidly opened until maximum opening and stayed at maximum GOA until rapidly closing shortly before full closure (Fig. 3E).

**Figure 4:**
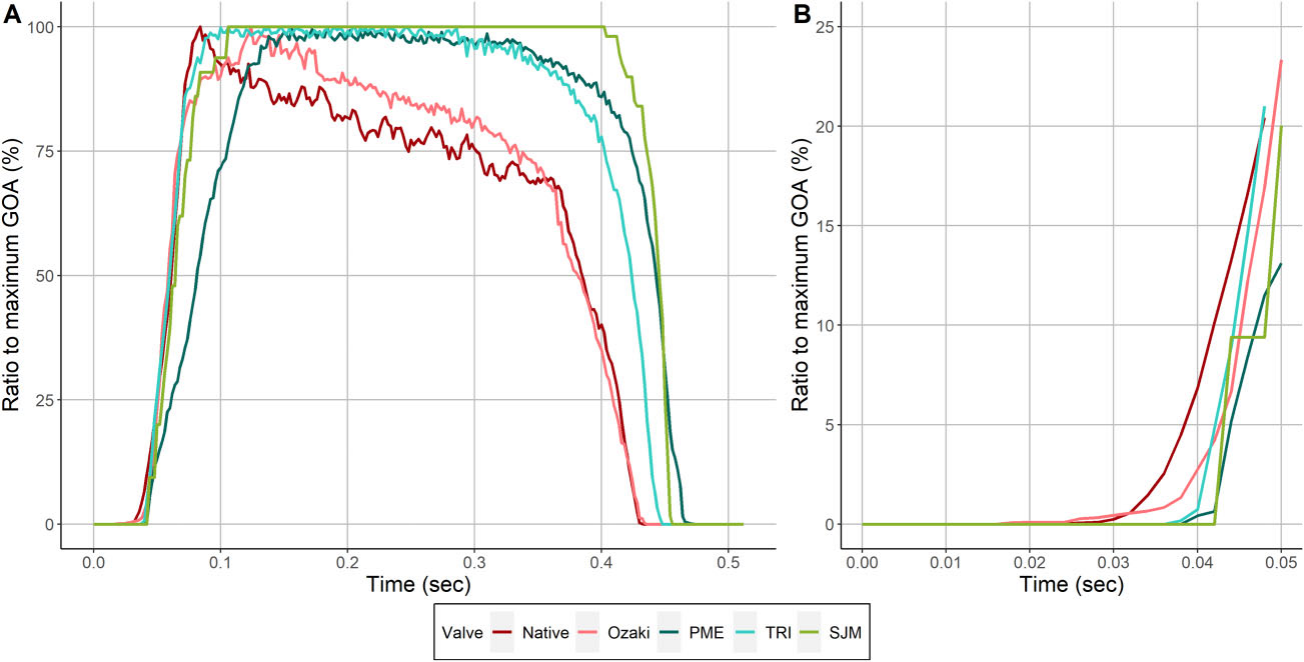
Overlay of GOA-flow-time plots of all investigated valves. (**A**) The maximum GOA was normalized to 100% for all valves. (**B**) Detailed view of GOA-flow-time plots at the very beginning of valve opening.

Figure 4A shows an overlay of all GOA-time curves with the GOA normalized to the maximum GOA of each valve. It appears that 3 groups can be distinguished from this figure: the native aortic valve and the Ozaki valve behave similarly, while both bioprosthetic valves show similar characteristics, and finally, the mechanical valve, which behaves differently compared with the other 2 groups. Figure 4B shows a detailed view of the opening phase of this overlap graph. The native valve and Ozaki valve opened earliest, while the prosthetic valves opened slightly later.

## DISCUSSION

Driven by the unmet need of an adequate treatment option for non-elderly patients with AS, an aortic neocuspidization procedure was introduced by Ozaki *et al.* [8]. However, unlike prosthetic valves, this valve substitute has not undergone extensive *in vitro* testing prior to in-human applications. Therefore, we aimed to characterize the hydrodynamic performance as well as cusp motion characteristics of the Ozaki valve under defined conditions using a setup meeting ISO requirements and provide a comparison to the native aortic valve and established prosthetic valves. The main findings of this study can be summarized as follows:

1. The Ozaki valve showed an improved hydrodynamic performance compared with prosthetic valves and was comparable to the native aortic valve.
2. The Ozaki valve and the native aortic valve showed a similar response to increased flow through the valve which was clearly distinguishable from prosthetic valves.
3. Similarly, the Ozaki valve and native aortic valve showed striking similarities regarding cusp motion characteristics while prosthetic valves clearly behaved differently.

Our study showed that the Ozaki neocuspidized valve achieved a larger EOA compared with established prosthetic valves, including the most widely implanted bovine pericardial valve (PME), a bioprosthesis with externally mounted cusps (TRI) and a bileaflet mechanical valve (SJM). All 3 investigated prosthetic valves are considered valves that achieve satisfactory haemodynamic results [1, 3, 12]. Furthermore, the EOA of the Ozaki valve was not inferior to the native aortic valve. These findings are plausible because the Ozaki procedure does not make use of a stent frame [13]. Data of a recent clinical study support these findings [14]. This is likely related to the fact that the opening area extends almost all the way to the annulus in the native and Ozaki valve while stented valves (mechanical as well as bioprosthetic) are limited to the inner diameter of the stent [15]. This may help to prevent patient–prosthesis mismatch, which is a known risk factor for impaired long-term outcomes [16]. The observed differences between the Ozaki valve and prosthetic valves were not very large, but it was previously shown that an mPG reduction of as little as 5 mmHg was associated with a 10.4% reduction of heart failure at 15 years [17]. Such small changes in mPG seem to be associated with an additional relevant relief of afterload which may be beneficial for the left ventricular myocardium, especially in the long run and in small annuli. Therefore, every effort must be undertaken to achieve the best possible result in young patients with a long life expectancy. From a pure quantitative hydrodynamic point of view, the Ozaki procedure seems to perform better than the investigated prosthetic valves and almost restore physiologic EOA. These findings are in line with a recent clinical study in which the Ozaki procedure resulted in a patient–prosthesis mismatch rate of 0% in patients with small annuli [18].

To investigate how the valves perform under ‘exercise’ conditions, we analysed the relationship between EOA, mPG and forward flow through the valve. As expected, the EOA increased with forward flow in all investigated valves (Fig. 2). However, the native valve and Ozaki valve behaved very similarly both showing a similar increase in EOA while all 3 prosthetic valves showed a markedly weaker increase. This was accompanied by the finding that mPG stayed relatively low in the native aortic and Ozaki valves while especially the 2 bioprosthetic valves showed a strong increase with forward flow (Fig. 2). In line with these results, a recent echocardiographic study demonstrated that 1 year after surgical aortic valve replacement using prosthetic valves, 1/3 of patients displays elevated pulmonary filling pressures during exercise [19]. Our results indicate that the Ozaki valve may be able to maintain larger EOA and lower gradients than prosthetic valves during states of increased cardiac output, may increase the quality of life and reduce the cardiac morbidity. This is most likely related to annular elasticity, which is maintained in the Ozaki valve, while the sewing ring used for prosthetic valves completely stiffens the annulus [20–22]. Annular elasticity may be important for another reason as well: few experimental studies have characterized native aortic valve opening and closure dynamics which can be divided into 4 phases [23]: (i) initial valve opening phase, (ii) rapid valve opening phase, (iii) slow valve closing phase and (iv) rapid valve closing phase. These characteristic phases are thought to be the result of a highly complex interplay between different functional units of the aortic root which involves all structures adjacent to the aortic valve and is aimed at the generation of a maximal opening area while at the same time minimizing cusp stress [24, 25]. This mechanism has been proposed to substantially contribute to the excellent durability of healthy native semilunar valves [26] and the presence of annular dynamics has been suggested to be a central part of this interplay [23, 24]. Therefore, preservation of annular elasticity in the Ozaki valve may lead to more physiological cusp motion characteristics. To investigate this hypothesis, we computed ‘GOA-flow-time plots’ (Fig. 3). In our model, the native valve behaved exactly as described previously in multiple experimental studies: it showed a rapid opening phase followed by a slow closing phase, which began in early systole when flow through the valve was still increasing [23, 27]. The Ozaki valve showed similar cusp motion characteristics with a slow closure phase, which also began in early systole when forward flow was still increasing. In contrast, this feature was not observed in any of the 3 prosthetic valves. The overlay of all investigated GOA-flow-time plots shows that the native and Ozaki valve behave very similarly while the 3 prosthetic valves can be clearly distinguished from them (Fig. 4A). In addition, when looking at the initial valve opening phase, the native and Ozaki valves opened earliest whereas prosthetic valves open slightly later (Fig. 4B). Previous experimental studies demonstrated that early valve opening occurs before the onset of blood flow through the valve due to expansion of the aortic root [23, 27, 28]. This mechanism is thought to reduce leaflet stress and thus increase cusp durability. Our observation that the Ozaki valve opens similarly early as the native valve indicates that shear stress on the Ozaki cusps may be lower than in prosthetic valves and may be associated with improved long-term durability.

Taken together, our data indicate that the Ozaki valve displays many aspects of native aortic root dynamics while prosthetic valves behave differently. Harken *et al.* proposed 10 commandments of the ‘perfect’ prosthetic heart valve, which included the capability of permanent fixation and the ease of implantation [29]. While these characteristics are found in most current prosthetic heart valves, other features such as chemical inertness or complete closure during diastole are lacking. Regarding one of the most important attributes, our study showed that the Ozaki valve behaves much like the native aortic valve, not only from a quantitative but also from a qualitative hydrodynamic point of view, therefore ‘offering no resistance to physiologic flows’. Whether these observations translate to a real benefit for patients remains to be investigated but our data lay a solid foundation that merits further investigations of the Ozaki procedure and its role in the treatment of patients suffering from AS.

### Limitations

Our model using a mock circulation loop and an *ex vivo* swine heart was designed to best possibly mimic human conditions but cannot entirely replicate *in vivo* conditions. Therefore, results may not entirely be transferrable to clinical conditions. Second, we utilized physiological saline instead of blood, which differs in viscosity and may have affected results. The number of experiments may have been too low to adequately detect differences. The mathematical relationship between forward flow and EOA as well as mPG is uncertain and polynomial regression may not be the best model.

## CONCLUSIONS

The Ozaki procedure showed similar characteristics as the native aortic valve in terms of EOA and mPG, the response to increased flow through the valve as well as cusp motion characteristics whereas prosthetic valves showed a markedly different behaviour. These features of the Ozaki valve may contribute to improved long-term durability of the valve and may therefore become a feasible treatment option in non-elderly adults.

## SUPPLEMENTARY MATERIAL

Supplementary material is available at *ICVTS* online.

## Funding

This work was supported by the German Heart Foundation/German Foundation of Heart Research (Dr. Rusche-Forschungsprojekt).

**Conflict of interest:** none declared.

## Supplementary Material

ivac199_Supplementary_Material

## Data Availability

The data underlying this article will be shared on reasonable request to the corresponding author.

## ABBREVIATIONS

AS: Aortic stenosis
EOA: Effective orifice area
mPG: Mean pressure gradient
PME: Perimount Magna Ease
SJM: St. Jude Medical Masters HP
TRI: Trifecta
