# Supplementary material for: Ex vivo evaluation of the Ozaki procedure in comparison with the native aortic valve and prosthetic valves
Source: Interact Cardiovasc Thorac Surg. 2022 Jul 27;35(3):ivac199. doi: 10.1093/icvts/ivac199 (PMC9443990; doi:10.1093/icvts/ivac199)
Supplement: ivac199_Supplementary_Material [file ivac199_Supplementary_Material.docx]

**Supplementary Table S1 –** Physical dimensions of investigated prosthetic valves according to the EACTS/STS/AATS Valve Labelling Task Force

| **Valve model** | **Labelled size** | **Physical dimensions** | | | | | | |
| --- | --- | --- | --- | --- | --- | --- | --- | --- |
|  |  | **Overall profile height** | **Outflow profile height** | **Minimum internal diameter** | **Internal stent diameter** | **External stent diameter** | **External housing diameter** | **External sewing ring diameter** |
| Magna Ease | 21 | 15 | 15 | 15 | 19 | 24 |  | 26 |
| Trifecta | 21 | 15 | 15 | 16 | 21 | 23 |  | 27 |
| SJM | 21 | 10 | 7 | 14.5 |  |  | 19 | 26 |

**Supplementary Table S2 –** Pairwise comparison of effective orifice area (EOA) and mean pressure gradient (mPG) of aortic valve substitutes under different hydrodynamic conditions. Bonferroni-Holm adjusted p-values are shown.

**A** Condition 1 (mean stroke volume 55.9 (standard deviation 2.3) ml)

Effective orifice area

|  | Native | Ozaki | PME | TRI |
| --- | --- | --- | --- | --- |
| Ozaki | 0.75 |  |  |  |
| PME | 0.20 | 0.28 |  |  |
| TRI | 0.023 | 0.046 | 0.75 |  |
| SJM | 0.20 | 0.37 | 1.00 | 0.43 |

Mean pressure gradient

|  | Native | Ozaki | PME | TRI |
| --- | --- | --- | --- | --- |
| Ozaki | 0.58 |  |  |  |
| PME | 0.059 | 0.252 |  |  |
| TRI | 0.059 | 0.26 | 0.97 |  |
| SJM | 0.50 | 0.58 | 0.33 | 0.33 |

**B** Condition 2 (mean stroke volume 73.3 (standard deviation 2.9) ml)

Effective orifice area

|  | Native | Ozaki | PME | TRI |
| --- | --- | --- | --- | --- |
| Ozaki | 0.47 |  |  |  |
| PME | 0.017 | 0.042 |  |  |
| TRI | 0.010 | 0.019 | 0.97 |  |
| SJM | 0.042 | 0.21 | 0.47 | 0.28 |

Mean pressure gradient

|  | Native | Ozaki | PME | TRI |
| --- | --- | --- | --- | --- |
| Ozaki | 0.58 |  |  |  |
| PME | 0.010 | 0.047 |  |  |
| TRI | 0.010 | 0.113 | 0.85 |  |
| SJM | 0.27 | 0.37 | 0.21 | 0.152 |

**C** Condition 3 (mean stroke volume 89.7 (standard deviation 3.3) ml)

Effective orifice area

|  | Native | Ozaki | PME | TRI |
| --- | --- | --- | --- | --- |
| Ozaki | 0.59 |  |  |  |
| PME | 0.003 | 0.011 |  |  |
| TRI | 0.003 | 0.011 | 0.97 |  |
| SJM | 0.039 | 0.039 | 0.176 | 0.140 |

Mean pressure gradient

|  | Native | Ozaki | PME | TRI |
| --- | --- | --- | --- | --- |
| Ozaki | 0.58 |  |  |  |
| PME | 0.005 | 0.019 |  |  |
| TRI | 0.010 | 0.028 | 0.58 |  |
| SJM | 0.40 | 0.53 | 0.034 | 0.126 |

**D** Condition 4 (mean stroke volume 104.1 (standard deviation 4.0) ml)

Effective orifice area

|  | Native | Ozaki | PME | TRI |
| --- | --- | --- | --- | --- |
| Ozaki | 0.70 |  |  |  |
| PME | 0.001 | 0.003 |  |  |
| TRI | 0.001 | 0.003 | 0.094 |  |
| SJM | 0.020 | 0.046 | 0.061 | 0.272 |

Mean pressure gradient

|  | Native | Ozaki | PME | TRI |
| --- | --- | --- | --- | --- |
| Ozaki | 0.72 |  |  |  |
| PME | 0.003 | 0.005 |  |  |
| TRI | 0.080 | 0.015 | 0.59 |  |
| SJM | 0.59 | 0.76 | 0.080 | 0.053 |

**Supplementary Figure S1 –** Still images of high speed video recordings at the corresponding time points during the cardiac cycle.

**
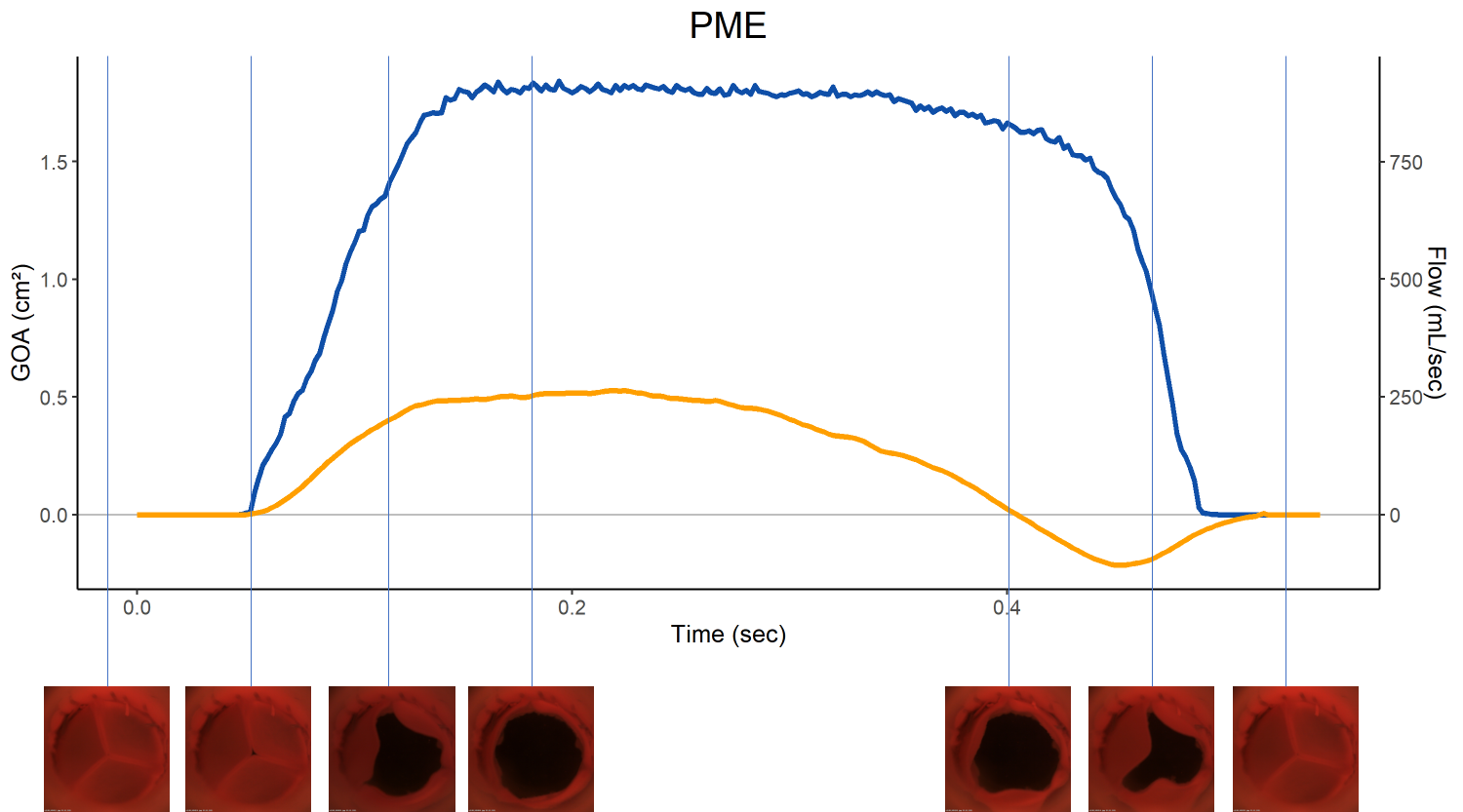
**
